# Validation of a Satisfaction Questionnaire on Spa Tourism

**DOI:** 10.3390/ijerph18094507

**Published:** 2021-04-23

**Authors:** Rosa Anaya-Aguilar, German Gemar, Carmen Anaya-Aguilar

**Affiliations:** Department of Economics and Business Administration, University of Malaga, Campus El Ejido, 29071 Malaga, Spain; ranaya@uma.es (R.A.-A.); canaya@uma.es (C.A.-A.)

**Keywords:** spas, satisfaction, tourism, health, factor analysis

## Abstract

The authors’ line of research is within the existing methodological debate around the concepts of quality of services, destinations, and quality measurements methods. The authors consider that the most appropriate way to measure quality is to develop instruments according to the destination and context in question, defining the quality of the tourist destination for practical purposes based on the satisfaction experienced by the tourist or the SERVPERF model, weighted and used to measure the quality of sun and beach tourist destinations. The authors of this work propose the knowledge of spa tourism, its quality and its level of satisfaction as a research gap and consider it as a starting point to validate a questionnaire that would allow the measurement and comparison of parameters with other segments already studied and that can also serve as a measuring instrument for tourist segments with similar characteristics, not as well known in the international literature as inland, ecological or nature tourism. Good internal reliability results were obtained in all items and in all dimensions. The factor analysis distributed the weights of the variables in the theoretical model, and construct validity was obtained with an association between the global evaluation by dimension and the general significance. The score of the main questionnaire was statistically significant.

## 1. Introduction

The evolution of research in medical tourism includes different groups of topics, such as issues related to health, well-being, thermal tourism, and quality of service, as well as topics related to medical treatments and tourism [1]. The basis for the implementation of a quality system in any company is the strategic quality plan. This involves objectively evaluating the current situation and contemplating the client’s vision [2,3]. There is a flood of definitions about the concept of quality, suggesting that it is a broad and multidimensional term with different interpretations. Quality has been defined from perspectives that are, in some cases, complementary (objective quality versus subjective quality; internal quality versus external quality) and, in other cases, antagonistic (static quality versus dynamic quality; absolute quality versus relative quality). Summarizing the literature, six concepts of quality have been identified [4]:

- Quality as excellence;
- Quality as conformity with specifications;
- Quality as uniformity;
- Quality as fitness for use;
- Quality as satisfaction of customer expectations;
- Quality as value creation, understood as the degree of satisfaction of all the key stakeholders of the organization or total quality.

Intuitively, a relationship can be determined between customer opinion and future results. Measuring perceived quality is “listening” to the customer and using it as a tool to organize opinions and determine areas for improvement on which to act. Quality is a multidimensional concept that encompasses many independent attributes (any item that is part of the definition), and the generic grouping of attributes defines the dimension. The analysis of perceived quality is a highly topical subject and an open field of research [4].

In relation to tourism experiences with water, some authors find a positive correlation with the perception of the quality of life, satisfaction and loyalty of people, both towards the experience and towards the destination [5].

Andalucía (Spain) is a region in Southern Spain with 87,268 km^2^ [6] and with a population of 8 million people [7], and it is well known as one of the main tourist destinations for sun and beach (e.g., Costa del Sol, Marbella or Malaga) and for cultural tourism (e.g., Córdoba, Granada and Alhambra) [8], in which young tourists and foreigners are predominate in summer and retirees constitute the majority in winter [9,10,11], but spas have not been extensively studied [12]. Therefore, the knowledge of spa tourism, its quality and level of satisfaction is considered in the present work as a research gap, and it is proposed as a starting point to validate a questionnaire that would enable measuring and comparing parameters with other segments and similar studies [13] and methodological approaches [13,14,15,16]. It is also interesting that it can serve as a measuring instrument for tourist segments with similar characteristics not so well known in the international literature, such as inland, ecological or nature tourism [17].

## 2. Literature Review

Many methodologies based on questionnaires establish theoretical satisfaction as a response to quality services, and there is a broad debate on the advantages and disadvantages of the use of each. Likert-type scales are easy to construct [18] and have high reliability and validity [15,19].

The debate stems from the fact that to fully measure the image of the destination, several components must be captured. These include attribute-based imagery; holistic impressions; and functional, psychological, unique, and common characteristics that require a combination of structured and unstructured methodologies to measure the image of the destination as envisioned in the conceptual framework [18].

Sasser et al. [20] consider that expectations result in attributes. Grönroos [21], representative of the Nordic model, states that the client compares the expected service with the service received. Parasuraman et al. [22,23], representatives of the American school, consider that to determine quality, it would be necessary to compare the difference between perceptions and expectations, and they elaborated a scale, SERVQUAL, with five dimensions. Cronin and Taylor [14] establish quality based solely on perceptions; that is, it eliminates the measurement of expectations but maintains the SERVPERF scale created by Parasuraman et al. [22,23]. Rust et al. [24] consider only three dimensions: result, delivery, and service environment. Dabholkar et al. [25] propose a hierarchy of quality in three levels or primary dimensions, and these, in turn, are divided into sub-dimensions. Brady and Cronin [26] consider dimensions, sub-dimensions, and level: reliability, responsiveness, and empathy. Therefore, the lack of consensus means that there are different models to assess quality.

The first aspect that arises when evaluating the quality of services is the measurement of expectation, which has been modeled on two widely used scales, SERVQUAL, which compares expectations and perceptions [27], and SERVPERF based solely on the measurement of perceptions [28]. Some authors doubt its validity due to theoretical difficulties and practical application measuring expectations [28]; others consider that the comparison between expectations and perceptions of quality is the most appropriate way to measure it [27]. Alén [29] makes a comparison of scales for the measurement of perceived quality in thermal establishments in which he concludes that the SERVPERF scale based on perceptions has better psychometric properties than the one based on the subtractive paradigm (perceptions–expectations).

Another point of debate is whether, when evaluating quality, the importance that users attach to each attribute or dimension should be measured. Cronin and Taylor [27] deduced from the results of their research that weighted models are different for studying the quality of services. Teas [30] makes a comparison between weighted and unweighted models and concludes that the latter are more appropriate in statistical terms. Quester et al. [31] make a comparison between weighted and unweighted models and conclude that the predictive power of SERVQUAL and SERVPERF improves with the inclusion of importance scores. On the other hand, among those in favor of including importance scores, there are also disagreements about how they should be measured. Parasuraman et al. [28] believe that scores should only be obtained for each factor [28], while others [31] argue that the importance should be asked individually for each attribute. In addition, there is a debate about whether importance scores should be obtained directly by asking the respondents [22] or if, on the contrary, they must be obtained indirectly through statistical procedures [13] from another type of information collected in the questionnaires.

The diversity of authors who have tried to define the tourist destination construct [4,32,33,34,35,36] indicates that the concept of tourist destination encompasses a great diversity of services offered by both private companies and public administrations, and even infrastructure and natural resources. Therefore, to evaluate the quality of a tourist destination, the methodologies developed in the field of quality can be applied, although taking into account that it is necessary to adapt them to a specific context [13].

Kozak and Rimmington [11] in Mallorca followed the line of measuring quality by developing and validating a destination measurement instrument composed of a multiattribute questionnaire, and according to Robinson [37], the best way to measure quality is by adapting the different instruments to the context. Otero and Otero [13], on Costa del Sol, used a multiattribute questionnaire that they validated using the CFA technique and developed a model to measure the quality of the tourist destination. They found high levels of satisfaction with the different attributes of the destination and other investigations obtained results along the same line [11,38,39,40,41].

## 3. Materials and Methods

### 3.1. Questionnaire Reliability: Test–Retest and Internal Reliability

A cross-sectional survey design was carried out, and the validated questionnaire by Otero and Otero [13], which in turn was inspired by the SERVPERF methodological school [28] study, was the basic instrument to obtain the sample information. The cultural adaptation to the specific spa tourism was carried out based on the contribution of a group of experts made up of university professors, professionals from the sector, and regular users of the spas. The result was a questionnaire with slight modifications to the original that serves to measure a complex construct, such as satisfaction in spa tourism or in segments with similar conditions, for which we have provided an operational definition by meeting with experts. Content validity could be obtained by following Streiner and Norman [42].

Under the previous premises, it was considered a priori that the questionnaire was made up of 7 factors or dimensions, which was later confirmed using the statistical technique of Confirmatory Factor Analysis (*n* = 725).

Once the adaptation was made, the diagnostic test–retest reproducibility test was carried out on 30 users (12 men and 18 women, aged 25–58 years), who did not belong to the main sample, and three study spas among the cases with a valid response with a separation between one and two days. The intraclass correlation coefficient (ICC) or Kappa index was used depending on the type of variable (quantitative or qualitative), valued with the Landis and Koch scale [43]: <0.00: poor (poor); 0.00–0.20: slight (slightly); 0.21–0.40: fair (fair); 0.41–0.60: moderate (moderate); 0.61–0.80; substantial (substantial); 0.81–1.00: almost perfect (almost perfect).

The internal consistency of the 52-item questionnaire was measured with Cronbach’s alpha coefficient excluding the item, which indicates that the questions that measure the same phenomenon are correlated with one another. The global alpha was obtained for each of the dimensions (accommodation, restoration, spa, sports facilities, leisure/culture/shopping, public roads/urban and natural environment, and transport infrastructure and other services) and by item in relation to its dimension.

The literature suggests that the Likert-type and semantic differential scales are easy to construct and manage [18]. The results of the empirical research show that the Likert-type and semantic differential scales have high reliability and validity [19]. The use of the “delighted–terrible scale” has been reported as the possibility of reducing satisfaction responses [15,16]. “Do not know” was also included in the scale of these possibilities for those who might not have an opinion due to no direct experience with the destination attribute. To obtain a weighting factor, the user was asked to grant a scale score of 1 to 100 on how important each factor was to him or her. The third section of the questionnaire, made up of four questions, was designed to determine overall satisfaction with the destination with the same seven-point scale, the intention to recommend it with three categories, and the intention to return with five, in which the user was only given asked by perceptions.

### 3.2. Data Collection Procedures

The sample was designed based on information gathered from external sources, in particular, a telephone and/or email survey of individuals responsible for managing spas. The relevant data were used to calculate the number of users per year per spa, as well as their distribution by age group and length of stay. The overall population size was estimated at 53,231 users per year with an age equal to or greater than 15 years old. These individuals were thus defined as this study’s primary subjects.

Stratified sampling was used, with each stratum coinciding with one of the participating spas. The sample size was estimated using Equation (1):

(1) $$n_{c}=Deff\times \;n$$

in which $n_{c}$ is the size adjusted to ensure complete sampling (i.e., strata) and Deff is the design’s effect or the relationship between the variance under stratified sampling and under simple random sampling. This variance was arbitrarily estimated a priori as 1.5, which was shown to be an overestimate. Any proportion calculated in this study had to meet the requirements of an accuracy of 5% and a 95% confidence level, with the worst possibility considered to be *p* = 0.5. This implied that *n* = 384 and nc = 576, which was set as the minimum.

The sample size and/or stratum was the result of a proportional fixation (i.e., the largest spas received more than one visit during the survey). The final result was 725 valid questionnaires, which coincides with some authors’ suggestion that sample size [44] be based on 10 subjects per analyzed variable. The sample size’s adequacy was thus confirmed, since 500 subjects would be an acceptable sample size and 1000 or more subjects would be an excellent sample size.

### 3.3. Factor Analysis, Calculation of Dimensions, and Validity of the Construct

In the factorial model of Otero and Otero [13] applied to sun and beach tourism, seven dimensions were obtained (accommodation, restaurants, beaches, sports facilities, leisure/culture/shopping, public roads/urban and natural environment, and transport infrastructures). In the present study, CFA was carried out to determine the degree to which the group of factors identified for sun and beach tourism is capable of representing the data from the spa tourism matrix.

To obtain a model with parsimony—that is, to maximize the amount of variance of the variables that can be explained by the minimum underlying factors or components—the Cattell slope test was performed, which consists of graphically representing the extracted factors (placed on the abscissa axis) against their eigenvalues (placed on the ordinate axis) to establish an inflection point on the graph. To obtain a more easily interpretable grouping of variables, the orthogonal rotation of factors was performed using the Varimax method, as it is the most frequently used [3,45]. To calculate the weight of each dimension, weights derived from the importance that each user gave to each of them were constructed, so that in each user, it would add up to 100 with all the weights in such a way that the weighting given by the *i-th* user was defined as follows:

$$w_{ij}=\frac{I_{ij}}{\sum_{J=1}^{7}I_{ij}}\times 100\;$$

where $I_{ij}$ is the importance given by use *i* to factor *j*. 

With this definition, the sum of the weights for each individual should be equal to 100.

$$\sum_{J=1}^{7}w_{ij}=100$$

The weight variable multiplies the value of each dimension to estimate the total score. The percentage distribution was not calculated for the weights, since it is the means that are relevant, taken from Otero and Otero [13]. The score of the items of global assessment by dimension and general satisfaction in users (*n* = 725) corresponded to the single question by dimension and global satisfaction questions and are different from the score by dimension estimated from the main questionnaire of 52 items.

The internal consistency of the questionnaire was complemented with questions of its future intention. A priori, it was to be expected that the response of intention to recommend it and intention to return would be related to the level of satisfaction, as observed in the satisfaction study of sun and beach tourism [10]. To determine the association between global assessment by dimension and general satisfaction and the score of the main questionnaire, the Pearson correlation coefficient was calculated. 

To calculate the association between the variables of future intention and the scores estimated in the main questionnaire (52 items and 725 users), the comparison of means was used. The aim was to check whether the difference between the two means was due to satisfaction influencing the intention to recommend it and the intention to return, or if the observed differences could simply be due to random variability. For this, the Student’s t test was applied for independent samples, with weighted samples. The null hypothesis was that both means are equal in the population. The alternative hypothesis maintained that the means were different and that both had different effects.

In all statistical analyses, the weighting of the observations was taken into account. For descriptive statistics, the SPSS Windows 15.0 program (SPSS Inc., Chicago, IL, USA) was used, and for analytical statistics (calculation of *p* values, as well as confidence intervals), the SUDAAN 7.0 program (RTI, RTP, Durham, NC, USA), indicating the STRWR sample design (stratified with replacement, the conglomerates being the strata). It was not corrected for a finite population, since the sample fraction (ratio between sample size and population) was much less than 10%. All the statistical procedures used, both those discussed in terms of reliability and validity and those carried out to evaluate the association of sociodemographic variables and characteristics of the type of visit by tourists to spas, are expressed in detail at the bottom of each results table.

## 4. Results

### 4.1. Sociodemographic Description of the Users and the Visit to the Spa 

The average age of spa tourists in Andalusia is 56 years old, and the tourists are predominantly female. Fifty-three percent of users are retired, and 34% are employed. Moreover, 29.8% have an average monthly income of EUR 500, 33.1% have an average monthly income between EUR 501 and 1000, and 26.6% are in the interval between EUR 1001 and 1500 (Table 1). The origin of these users is mainly local, with 82.9% from the Andalusian community, with no foreigners in the sample (Table 2). In addition, 51.3% of spa tourists in Andalusia go as a couple and 19.7% as a family. In the accommodation modality, 49.6% choose a three-star hotel. The highest percentage of overnight stays is among the IMSERSO (48.4%), followed by weekend and long-weekend tourists (28.1%), with an average stay of one week. Moreover, 46.8% of users who go to a spa repeat their visit, contracting the trip without intermediaries (99.1%) (Table 1).

At the population level, 92.4% express the intention of recommending the spa compared to 2.0% who have no intention of recommending it. Additionally, 63.6% of the population express the intention to return next year compared to 1.6% who have no intention of ever returning (Table 2).

### 4.2. Validation of the Satisfaction Questionnaire in Spa Tourism

#### 4.2.1. Test–Retest

For the diagnostic agreement of the 52 items of the main questionnaire, a test–retest was applied (with a separation between 1 and 2 days), analyzed with the Intraclass Correlation Coefficient (ICC), valued with the Landis and Koch scale (39): <0.0: poor; 0.00–0.20: slight; 0.21–0.40: fair; 0.41–0.60: moderate; 0.61–0.80: substantial; 0.81–1.00: almost perfect.

To calculate the diagnostic concordance of the 52 items of the main questionnaire, the test–retest (with a separation between one and two days), the Intraclass Correlation Coefficient (ICC) was used, assessed with the Landis and Koch scale [43]: <0.0: poor; 0.00–0.20: slight; 0.21–0.40: fair; 0.41–0.60: moderate; 0.61–0.80: substantial; 0.81–1.00: almost perfect.

Diagnostic concordance was found with scores higher than 0.65 for almost all the items of the accommodation dimension. In the restoration dimension, values higher than those of accommodation were found, mostly above 0.67; when analyzing the spa dimension, we found values higher than 0.51; in the sports facilities dimension, these values were above 0.67; regarding leisure/culture/shopping, values higher than 0.61 were obtained; for the dimension of public roads, and urban and natural environments, the values were in almost all respects above 0.36; and finally, for the dimension of transport, infrastructures and other services, the CCI value was higher in all variables than 0.61 (Table 3).

In the general assessment questions (global assessment by dimension, importance, general satisfaction, satisfaction in quality/price, intention to recommend it, and intention to return), an ICC of greater than 0.61 was obtained in all the items of the quantitative variables and a kappa index higher than 0.70 for the qualitative ones (Table 4).

**Table 4:** Reliability of the questionnaire. General valuation questions. Test–retest with a separation between one and two days *n* = 30.

Question	Initial	Repetition (24–48 h.)	Concordance ^a^
Global assessment by dimension			
Accommodation	5.87 ± 1.25	5.77 ± 1.10	ICC = 0.75
Catering (meals)	5.83 ± 0.99	5.53 ± 1.01	ICC = 0.46
Spa	5.50 ± 0.90	5.37 ± 1.03	ICC = 0.50
Sports facilities	4.53 ± 1.96	4.43 ± 1.77	ICC = 0.83
Leisure, culture, and shopping	4.37 ± 1.40	4.43 ± 1.65	ICC = 0.66
Urban and natural environment	5.27 ± 1.23	5.07 ± 1.34	ICC = 0.70
Infraest. transport and other serv.	4.43 ± 1.43	4.93 ± 1.36	ICC = 0.67
Importance of the factor (mean ± of ^b^)			
Accommodation	85 ± 18	87 ± 13	ICC = 0.62
Catering (meals)	80 ± 22	80 ± 14	ICC = 0.61
Spa	80 ± 24	76 ± 18	ICC = 0.35
Sports facilities	45 ± 24	50 ± 22	ICC = 0.68
Leisure, culture, and shopping	59 ± 21	61 ± 21	ICC = 0.73
Urban and natural environment	76 ± 22	77 ± 20	ICC = 0.85
Infraest. transport and other serv.	58 ± 23	60 ± 23	ICC = 0.77
General stay satisfaction (mean ± de)	5.97 ± 1.03	5.90 ± 0.99	ICC = 0.81
Satisfaction in quality/price (mean ± de)	5.83 ± 1.09	5.77 ± 0.94	ICC = 0.71
Intention to recommend it (%)			kappa ^c^ = 1
Yes	93.3	93.3	
No	3.3	6.7	
Does not know	3.3	0.0	
Intention to return (%)			kappa = 0.70
Next year	13.3	13.3	
This year	13.3	10.0	
Ever	43.3	60.0	
Does not know	26.7	13.3	
Never	3.3	3.3	

^a^: Measured by ICC or kappa, depending on the type of variable. Both ICC and kappa are interpreted with the Landis and Koch scale (see footnote d in Table 5). ^b^: Arithmetic mean ± SD unweighted. ^c^: The kappa was calculated after eliminating the case (3.3%) that did not answer in the initial assessment.

**Table 5:** Internal reliability of the questionnaire (Cronbach’s alpha) (*n* = 725) ^a^.

By Dimension				By Item (in Relation to Its Size)		
Dimension	No. Items	*n* ^b^	Global Alpha	Item	Corrected Item- Total Correlation	Alpha without Item
Accommodation	8	636	0.871	01-Cleaning, hygiene	0.756	0.842
				02-Security	0.688	0.850
				03-Environmental quality	0.556	0.862
				04-Room	0.689	0.848
				05-Aesthetics	0.730	0.843
				06-Ease for children and disabled	0.389	0.892
				07-Personal professionalism	0.688	0.850
				08-Value for money	0.667	0.851
Catering	6	710	0.919	09-Variety	0.823	0.897
				10-Cleaning	0.776	0.904
				11-Environment	0.664	0.918
				12-Food quality	0.840	0.894
				13-Personal professionalism	0.696	0.914
				14-Quality/price ratio	0.829	0.896
Spa	9	682	0.833	15-Medicinal mineral waters	0.577	0.817
				16-Cleaning	0.669	0.808
				17-Security	0.541	0.820
				18-Equipment	0.601	0.809
				19-Environment	0.579	0.812
				20-Medical service	0.513	0.819
				21-Handicapped access	0.425	0.851
				22-Personal professionalism	0.645	0.805
				23-Value for money	0.645	0.804
Sports facilities	6	520	0.907	24-For children	0.533	0.917
				25-Various	0.694	0.898
				26-Soft (paddle, etc.)	0.795	0.883
				27-Tennis	0.835	0.877
				28-Pool	0.730	0.893
				29-Others	0.879	0.869
Leisure/culture/shopping	7	562	0.830	30-Serv. complem. (parking)	0.440	0.828
				31-Pub/discos/cafes	0.780	0.775
				32-Excursions	0.600	0.803
				33-Culture/show	0.690	0.789
				34-Shops	0.786	0.773
				35-Animation of streets	0.491	0.820
				36- Tourist office	0.357	0.853
Public roads/urban and natural environment	9	713	0.869	37-Cleaning	0.585	0.857
				38-Absence of beggars	0.623	0.857
				39-Feeling of security	0.705	0.847
				40-Little noise	0.627	0.853
				41-Traffic	0.683	0.847
				42-Urban furniture	0.560	0.863
				43-Attitude of the people	0.527	0.861
				44-Urban landscape	0.668	0.848
				45-Natural environment	0.575	0.858
Transport infrastructures and other services	7	542	0.803	46-Taxis	0.309	0.816
				47-Buses	0.484	0.787
				48-Roads	0.449	0.793
				49-Line buses	0.571	0.771
				50-Post office	0.710	0.742
				51-Public telephones	0.579	0.770
				52-Banking services	0.660	0.753

^a^: Note that it is a weighted sample. ^b^: Number (unweighted) of users out of the total of 725 who responded to all items in the dimension.

#### 4.2.2. Internal Reliability

The internal consistency of the questionnaire measured with Cronbach’s alpha coefficient indicates a high correlation between the items. The global values for accommodation were at 0.871; restoration: 0.919; spa: 0.833; sports facilities: 0.907; leisure/culture/shopping: 0.830; public roads/urban and natural environment: 0.869; and transport infrastructure and other services: 0.803 (Table 6).

By item (in relation to its dimension), we found a corrected item–total correlation within the accommodation dimension for “cleanliness and hygiene” of 0.756 and for “aesthetics” of 0.730 as the highest values. The lowest value was obtained by “facility for children and the disabled” at 0.389. For the restoration dimension, the value was 0.840 for “food quality” and 0.829 for “value for money” as high values, and 0.664 for “environment” as the lowest value. For the spa dimension, these values were between 0.669 for “cleanliness” and 0.425 for “disabled access”. In the sports facilities dimension, we obtained 0.879 for “other sports facilities” and the lowest value of 0.533 for “children”. In the case of the leisure/culture/shopping dimension, the highest value was 0.786 for “shops”, and the lowest value was 0.357 for “tourist office”. In public roads/urban and natural environment, we obtained 0.705 for “sense of security” and 0.527 for “people’s attitude”. Finally, in the case of the dimension of transport infrastructures and other services, the highest value was 0.710 for “post office”, and the lowest values were 0.309 for “taxis” and 0.449 for “roads” (Table 5).

#### 4.2.3. Factor Analysis and Calculation of Dimensions

The KMO test, which compares the Pearson correlation coefficients between each pair of variables with their respective partial correlation coefficients and whose values oscillate between 0 and 1, obtained an excellent value of adequacy of the weighted sample, KMO = 0.923 (Table 6). The Bartlett sphericity test, whose null hypothesis is that there is no correlation between variables, obtained a *p* value <0.001, which rejects the null hypothesis of the absence of correlation between variables, which indicates the adequacy of the CFA (Table 7).

**Figure 1:**
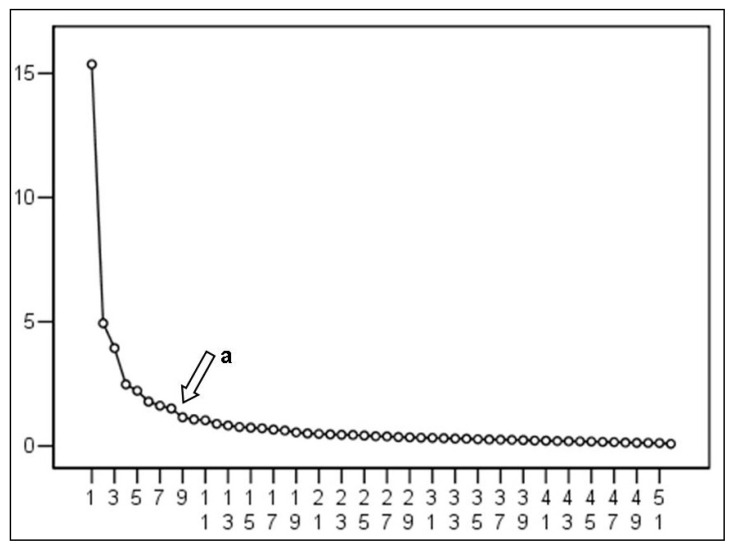
Cattel slope.

The Cattel slope test (Figure 1) indicates a number of factors to be extracted from eight factors, coinciding with the theoretical model (38).

After the Varimax orthogonal rotation, a grouping of variables was obtained that distributed the weights over the dimensions indicated in the theoretical model, with slight imbalances in the variables that distributed a similar weight in two different factors, such as the variables ease for children and the disabled, medical service, complementary services, people’s attitudes, and natural environment. The infrastructure and other services dimension was divided into two, leaving, on the one hand, roads and other services and, on the other, the variables referring to transport, such as taxis, urban buses, and line buses (Table 7). The variance explained with rotated factors (Varimax rotation) is shown in Table 8.

#### 4.2.4. Validity of the Construct

The association (Pearson correlation) between global assessment by dimension, general satisfaction and score of the main questionnaire (*n* = 725) of the weighted sample in all cases was statistically significant (*p* < 0.001). Pearson’s linear correlation coefficient r showed positive values close to 1, 0.91 for the restoration dimension and 0.69 for transport infrastructure and other services, which showed a close and direct association between them (Table 9).

Regarding the association (comparison of means) between the variables of future intention and the scores estimated with the main questionnaire of 52 items (*n* = 725) of the weighted sample, they are significant (*p* < 0.001) with respect to greater satisfaction (Table 10).

## 5. Discussion and Conclusions

The questionnaire had three structured sections for the purpose of measuring the satisfaction of spa tourists with general identification questions, specific questions of satisfaction, their probability of returning, and their probability of recommending the destination. The second section, made up of 52 items, was based on a seven-point scale from delighted to terrible, measuring seven factors, namely, accommodation, restaurants, spa, sports facilities, leisure/culture/shopping, public roads/urban and natural environment, and infrastructures and other services. 

Our research coincides with other studies that have identified specific attributes of the destination at a lower cost [11,13].

The reference model was taken into account and contrasted with the suggestions of the experts for adaptation to the context, finding a good general fit with the theoretical model and some particularities, such as the variables of ease for children and the disabled, medical service, complementary services, the attitude of the people, and the natural environment, which distributed their weight in two different factors and which the authors define as complex variables [46]. The dimension “infrastructures and other services” was divided into two, leaving, on the one hand, roads and other services and, on the other, the variables related to transport, such as taxis, urban buses, and scheduled buses. Otero and Otero [13] justify the categorization of the items based on the advantage of considering that the dimensions within several factors obtain more useful information to carry out quality policies, since it allows the identification of those responsible. On the other hand, it would be logical to disaggregate transport infrastructures and other services based on the practical knowledge acquired in this work. The CFA for five dimensions explains 48% of the total variance compared to the study by Otero and Otero [13], where it only explains 36%. However, in the analysis of the results with eight components, it explains 65.1% of the total variance.

All this questions the validity of the construct. However, the results of the global assessment obtained by dimension in the weighted sample corresponding to the single question per dimension of the questionnaire and different from the one estimated from the main questionnaire of 52 items, as well as the general questions regarding general satisfaction, stood out with higher average values. The study found statistical significance between the highest satisfaction values and the variables of future intention. Other investigations obtained similar results [11,13,32,39,40,41]. The validated questionnaire of satisfaction on sun and beach tourism in Costa del Sol is adapted to the spa sector, adjusting to the seven initial dimensions, except for transport infrastructures, which is divided into two, on the one hand, roads and public telephones, and on the other hand, taxis and buses.

The findings of this research show a solid instrument that can be used by different institutions to replicate and use as a barometer of spa tourism and culturally adapted to be used for similar segments. With this instrument, one can obtain both the level of satisfaction and the factors associated with it, and even segment the market.

The analysis is limited only to spas located in Southern Spain, so the results can be extrapolated to any spa in Spain or any country with similar climatic conditions.

## Figures and Tables

**Table 1:** Sociodemographic distribution of users (*n* = 725).

	Sample	Population
Variable	*n* (%)	% ± se ^a^
Age (years)		
15–35	137 (18.9)	18.1 ± 1.6
36–49	116 (16.0)	14.6 ± 1.5
50–64	149 (20.6)	21.2 ± 1.8
65–88	323 (44.6)	46.1 ± 2.1
Mean ± SD/se ^b^	55.2 ± 18.3	56.0 ± 0.8
Sex		
Male	286 (39.4)	38.2 ± 2.1
Woman	439 (60.6)	61.8 ± 2.1
Occupation		
Occupied	268 (37.0)	34.0 ± 2.0
Housewife	45 (6.2)	5.8 ± 1.0
Student (*n* = 48) or unemployed (*n* = 3)	51 (7.0)	7.0 ± 1.1
Retired	361 (49.8)	53.1 ± 2.1
Monthly income (euros)		
<500	207 (28.6)	29.8 ± 2.0
501–1000	229 (31.6)	33.1 ± 2.0
1001–1500	194 (26.8)	26.6 ± 1.9
501–2000	77 (10.6)	8.6 ± 1.1
2001–2500	11 (1.5)	1.0 ± 0.3
2501–3000	4 (0.6)	0.7 ± 0.4
>3000	3 (0.4)	0.2 ± 0.1
Region of origin ^c^		
Total Andalusia	596 (82.2)	82.9 ± 1.6
Canary Islands	2 (0.3)	0.1 ± 0.1
Cantabria	1 (0.1)	0.0 ± 0.0
Castilla Leon	2 (0.3)	0.1 ± 0.1
Castilla Mancha	13 (1.8)	2.6 ± 0.8
Catalonia	22 (3.0)	2.3 ± 0.5
Estremadura	3 (0.4)	0.2 ± 0.1
The Rioja	2 (0.3)	0.2 ± 0.1
Madrid	55 (7.6)	7.9 ± 1.1
Murcia	4 (0.6)	1.1 ± 0.6
Basque Country	2 (0.3)	0.2 ± 0.2
Valencia	16 (2.2)	1.7 ± 0.5
Ceuta/Melilla	7 (1.0)	0.5 ± 0.2
Andalusian Provinces (on national total)		
Almeria	73 (10.1)	8.3 ± 0.8
Cadiz	39 (5.4)	4.5 ± 0.8
Cordova	25 (3.4)	2.7 ± 0.6
Granada	127 (17.5)	18.3 ± 1.6
Huelva	5 (0.7)	0.9 ± 0.5
Jaen	104 (14.3)	20.4 ± 1.6
Malaga	178 (24.6)	21.9 ± 1.7
Seville	45 (6.2)	5.7 ± 1.0

^a^: Population percentage ± standard error, taking into account the weight of the sample and stratified sampling (Spas) with SUDAAN. ^b^ The standard deviation (SD) was calculated in the case of the sampling distribution and the standard error (se) in the population distribution. ^c^ In the sample, there were no users of foreign origin or from the regions of Aragon, Asturias, Balearic Islands, Galicia, or Navarra.

**Table 2:** Distribution of the variables of future intention of users (*n* = 725) ^a^.

	Sample	Population
Variable	*n* (%)	% ± se ^b^
Intent to recommend it		
Yes	665 (91.7)	92.4 ± 1.1
No	17 (2.3)	2.0 ± 0.6
Does not know	43 (5.9)	5.6 ± 1.0
Intention to return		
Next year	470 (64.8)	63.6 ± 2.0
This year	96 (13.2)	11.0 ± 1.1
Ever	93 (12.8)	16.0 ± 1.7
Does not know	54 (7.4)	7.7 ± 1.2
Never	12 (1.7)	1.6 ± 0.5

^a^ There is no casa in which they do not answer these questions. ^b^ Population percentage ± standard error, taking into account the weight of the sample and stratified sampling (Spas) with SUDAAN.

**Table 3:** Reliability of the 52 items of the main questionnaire. Test–retest (1–2 days apart) (*n* = 30 ^a^).

Dimension ^b^: Item–Item No.	Initial	Repetition (24–48 h.) Mean ± SD ^c^	ICC ^d^
ACC: 01-Cleaning, hygiene	5.90 ± 1.32	5.87 ± 1.20	0.80
ACC: 02-Security	5.67 ± 1.21	5.57 ± 1.30	0.65
ACC: 03-Environmental quality	5.87 ± 1.07	5.43 ± 1.19	0.65
ACC: 04-Room	5.63 ± 1.16	5.33 ± 1.18	0.46
ACC: 05-Aesthetics	5.37 ± 1.56	5.33 ± 1.42	0.66
ACC: 06-Facility for children and disabled	4.93 ± 1.74	4.87 ± 1.53	0.84
ACC: 07-Personal professionalism	5.57 ± 1.43	5.57 ± 1.04	0.74
ACC: 08-Quality/price ratio	5.77 ± 1.14	5.67 ± 1.03	0.67
CAT: 09-Variety	5.10 ± 1.27	5.17 ± 1.15	0.68
CAT: 10-Cleaning	5.90 ± 0.99	5.83 ± 0.91	0.67
CAT: 11-Environment	5.23 ± 1.33	5.17 ± 1.26	0.68
CAT: 12-Food quality	5.63 ± 1.07	5.43 ± 1.07	0.56
CAT: 13-Personal professionalism	5.90 ± 1.16	5.57 ± 1.04	0.71
CAT: 14-Ratio quality/price	5.77 ± 1.07	5.60 ± 1.16	0.53
SPA: 15- Medicinal mineral waters	6.00 ± 1.08	5.70 ± 1.26	0.51
SPA: 16-Cleaning	6.07 ± 1.05	5.93 ± 1.11	0.68
SPA: 17-Security	5.57 ± 1.01	5.83 ± 1.15	0.43
SPA: 18-Equipment	5.50 ± 1.04	5.20 ± 1.27	0.67
SPA: 19-Environment	5.33 ± 1.30	5.43 ± 1.07	0.51
SPA: 20-Medical service	4.87 ± 1.63	4.90 ± 1.40	0.64
SPA: 21-Handicapped access	4.40 ± 1.81	4.50 ± 1.59	0.67
SPA: 22-Personal professionalism	5.60 ± 1.13	5.27 ± 1.44	0.51
SPA: 23-Value for money	5.50 ± 0.90	5.30 ± 1.34	0.23
SPO: 24-For children	5.30 ± 1.49	5.27 ± 1.39	0.67
SPO: 25-Various	4.87 ± 1.59	4.87 ± 1.43	0.72
SPO: 26-Soft (paddle, etc.)	3.93 ± 1.82	4.23 ± 1.70	0.74
SPO: 27-Tennis	3.20 ± 2.11	3.14 ± 2.03	0.82
SPO: 28-Pool	5.30 ± 1.95	5.10 ± 1.77	0.81
SPO: 29-Others	4.13 ± 2.01	4.03 ± 1.99	0.84
LEI: 30-Serv. complem. (parking)	5.03 ± 1.83	5.17 ± 1.46	0.82
LEI: 31-Pub/discos/cafes	3.97 ± 1.47	4.10 ± 1.54	0.76
LEI: 32-Excursions	4.27 ± 1.72	4.37 ± 1.52	0.61
LEI: 33-Culture/shows	4.00 ± 1.72	4.20 ± 1.83	0.71
LEI: 34-Shops	4.43 ± 1.70	4.27 ± 1.76	0.65
LEI: 35-Street animation	4.43 ± 1.77	4.77 ± 1.52	0.61
LEI: 36-Tourist office	4.57 ± 1.77	4.90 ± 1.63	0.78
PUB: 37-Cleaning	5.50 ± 1.41	5.33 ± 1.37	0.73
PUB: 38-Absence of beggars	5.87 ± 1.31	5.37 ± 1.45	0.63
PUB: 39-Feeling of security	5.30 ± 1.37	5.10 ± 1.40	0.58
PUB: 40-Little noise	4.87 ± 1.41	4.57 ± 1.43	0.36
PUB: 41-Traffic	4.23 ± 1.70	4.60 ± 1.33	0.38
PUB: 42-Urban furniture	4.77 ± 1.50	4.93 ± 1.17	0.55
PUB: 43-Attitude of the people	5.80 ± 1.30	5.47 ± 1.43	0.67
PUB: 44-Urban landscape	5.10 ± 1.81	5.20 ± 1.45	0.64
PUB: 45-Natural environment	5.67 ± 1.30	5.47 ± 1.38	0.67
INF: 46-Taxis	3.93 ± 1.68	4.03 ± 1.50	0.73
INF: 47-Buses	3.93 ± 1.70	4.07 ± 1.46	0.73
INF: 48-Roads	5.07 ± 1.01	5.20 ± 1.10	0.64
INF: 49-Line buses	3.93 ± 1.78	4.63 ± 1.71	0.69
INF: 50-Post office	4.43 ± 1.79	4.70 ± 1.58	0.69
INF: 51-Public telephones	4.53 ± 1.50	4.63 ± 1.40	0.61
INF: 52-Banking services	4.60 ± 1.75	4.87 ± 1.43	0.71

^a^: Corresponds to 30 users, 12 men and 18 women, aged 15–88 years, not belonging to the main sample, and from three spas in the study. The concordance of the variables age, sex, occupation, income, transport used, and type of accommodation was virtually 100% (results not shown in the table). ^b^: ACC: accommodation; CAT: catering; SPA: spa; SPO: sports facilities; LEI: leisure/culture/shopping; PUB: public roads/urban and natural environment; INF: transport infrastructures and other services. ^c^: Arithmetic mean ± SD unweighted only with valid cases. ^d^: ICC between cases with valid response. It can be assessed with the Landis and Koch scale [43]: <0.00: poor; 0.00–0.20: slight (slightly); 0.21–0.40: fair (fair); 0.41–0.60: moderate (moderate); 0.61–0.80: substantial (substantial); 0.81–1.00: almost perfect (almost perfect). Based on 29 individuals, since one did not respond in the repetition.

**Table 6:** Diagnostic criteria of the factor analysis (*n* = 725) ^a^.

Criterion	Outcome
Kaiser–Meyer–Olkin (adequacy measure)	0.923
Bartlett’s sphericity test	χ2 = 18082.2 (1326 gl.), *p* < 0.001

^a^ Note that it is a weighted sample.

**Table 7:** Factor analysis (*n* = 725). Factors (8) after Varimax rotation, with all weights ^a^.

	Factors (from Factor Analysis) and Approximate Interpretation							
Questionnaire Dimension ^b^: Item-Item No.	1 ≈SPO	2 ≈ACC	3 ≈LEI	4 ≈SPA	5 ≈PUB	6 ≈CAT	7 ≈PUB	8 ≈INF
ACC: 01-Cleaning, hygiene	0.08	0.77	0.04	0.18	0.16	0.19	0.06	0.10
ACC: 02-Security	0.04	0.75	0.10	0.16	0.20	0.15	0.06	−0.06
ACC: 03-Environmental quality	−0.12	0.64	0.00	0.10	0.23	0.21	−0.06	0.13
ACC: 04-Room	0.19	0.68	0.06	0.17	0.10	0.15	0.03	0.20
ACC: 05-Aesthetics	−0.05	0.75	0.17	0.26	0.16	0.13	−0.01	0.09
ACC: 06-Facility for children and disabled.	0.55	0.34	0.21	0.28	−0.04	0.04	−0.19	−0.12
ACC: 07-Personal professionalism	0.22	0.66	0.05	0.14	0.19	0.28	0.06	0.00
ACC: 08-Quality/price ratio	0.24	0.58	0.07	0.31	0.02	0.28	0.11	0.04
CAT: 09-Variety	0.23	0.23	0.19	0.28	0.15	0.72	0.03	0.08
CAT: 10-Cleaning	0.08	0.29	0.13	0.28	0.20	0.70	0.01	0.12
CAT: 11-Environment	−0.02	0.35	0.05	0.25	0.33	0.57	0.07	−0.15
CAT: 12-Food quality	0.23	0.24	0.08	0.27	0.16	0.74	0.05	0.08
CAT: 13-Personal professionalism	0.15	0.34	0.07	0.09	0.17	0.69	0.12	−0.07
CAT: 14-Ratio quality/price	0.23	0.26	0.12	0.29	0.08	0.73	0.13	0.04
SPA: 15-Medicinal mineral waters	−0.07	0.27	0.06	0.60	0.19	0.23	0.12	−0.04
SPA: 16-Cleaning	0.02	0.31	0.04	0.65	0.16	0.22	0.10	0.08
SPA: 17-Security	0.02	0.28	0.07	0.43	0.31	0.24	0.15	−0.22
SPA: 18-Equipment	0.36	0.13	0.12	0.60	0.02	0.12	−0.10	0.17
SPA: 19-Environment	0.03	0.34	0.12	0.52	0.25	0.15	0.05	0.02
SPA: 20-Medical service	0.04	0.36	0.14	0.34	0.18	0.25	0.22	0.00
SPA: 21-Handicapped access	0.57	0.08	0.14	0.38	−0.04	0.17	−0.25	−0.01
SPA: 22-Personal professionalism	0.31	0.26	−0.17	0.55	0.10	0.19	0.10	0.04
SPA: 23-Value for money	0.31	0.19	−0.03	0.61	0.01	0.21	0.11	0.09
SPO: 24-For children	0.55	0.10	0.32	0.28	0.03	0.41	−0.12	−0.01
SPO: 25-Various	0.76	0.05	−0.10	0.33	0.01	0.02	−0.06	0.07
SPO: 26-Soft (paddle, etc.)	0.79	0.06	0.10	0.04	−0.03	0.08	0.21	0.09
SPO: 27-Tennis	0.80	−0.03	0.08	−0.10	0.06	0.15	0.26	0.06
SPO: 28-Pool	0.76	0.07	−0.17	−0.06	0.21	0.11	−0.10	0.13
SPO: 29-Others	0.84	0.00	0.11	−0.06	0.03	0.14	0.20	0.01
LEI: 30-Serv. complem. (parking)	0.51	0.40	0.36	0.12	0.02	0.03	0.05	0.02
LEI: 31-Pub/discos/cafes	0.01	0.12	0.87	0.05	0.05	0.09	0.10	0.03
LEI: 32-Excursions	0.27	0.16	0.61	−0.07	0.19	0.32	−0.04	0.27
LEI: 33-Culture/show	0.20	0.14	0.70	−0.05	0.13	0.30	0.06	0.21
LEI: 34-Shops	0.05	0.10	0.87	0.03	0.00	0.07	0.14	0.07
LEI: 35-Street animation	0.00	0.08	0.55	0.32	0.36	0.15	−0.03	0.17
LEI: 36-Tourist office	−0.18	0.02	0.59	0.14	−0.13	−0.15	0.23	0.10
PUB: 37-Cleaning	0.26	0.01	0.03	0.23	0.59	0.12	−0.02	0.22
PUB: 38-Absence of beggars	0.07	0.13	0.04	0.37	0.63	0.04	0.05	−0.16
PUB: 39-Feeling of security	0.03	0.22	0.14	0.19	0.74	0.11	0.08	−0.17
PUB: 40-Little noise	−0.14	0.29	−0.06	−0.05	0.75	0.12	−0.05	0.19
PUB: 41-Traffic	0.04	0.29	−0.07	−0.03	0.77	0.18	0.01	0.02
PUB: 42-Urban furniture	0.34	0.12	0.01	0.02	0.58	0.13	0.28	0.29
PUB: 43-Attitude of the people	0.03	0.16	0.24	0.46	0.43	0.16	−0.01	−0.08
PUB: 44-Urban landscape	−0.02	0.09	0.27	0.43	0.57	0.14	0.10	0.12
PUB: 45-Natural environment	−0.12	0.09	0.23	0.52	0.46	0.15	0.13	0.02
INF: 46-Taxis	0.06	0.15	0.22	0.10	0.17	0.01	−0.19	0.76
INF: 47-Buses	0.14	0.11	0.16	0.07	−0.07	−0.03	0.15	0.81
INF: 48-Roads	−0.09	0.11	0.20	0.18	−0.02	0.17	0.70	0.05
INF: 49-Line buses	0.06	0.10	0.23	−0.04	0.12	0.16	0.49	0.57
INF: 50-Post office	0.33	−0.04	0.61	0.13	0.05	0.02	0.54	0.07
INF: 51-Public telephones	0.30	0.12	0.27	0.10	0.27	−0.01	0.65	−0.01
INF: 52- Banking services	0.38	−0.06	0.55	0.13	0.07	0.13	0.47	−0.09

^a^: The largest weights in each variable are in bold. Note that it is a weighted sample. ^b^: ACC: accommodation; CAT: catering; SPA: spa; SPO: sports facilities; LEI: leisure/culture shopping; PUB: public roads/urban and natural environment; INF: transport infrastructures and other services.

**Table 8:** Variance explained with rotated factors (Varimax rotation) (*n* = 725) ^a^.

Factor	Interpretation Approximate	Sum of Saturations Squared after Rotation		
		Total	% Variance	% Accumulated
1	≈Sports facilities	5.7	11.0	11.0
2	≈Accommodation	5.2	10.1	21.1
3	≈Leisure, culture, and shopping	4.7	9.1	30.2
4	≈Spa	4.6	8.9	39.1
5	≈Public roads, urban and natural environment	4.6	8.8	48.0
6	≈Catering (meals)	4.3	8.2	56.2
7	≈Infraest. transp. and other serv. (roads, public phones) ^b^	2.4	4.6	60.8
8	≈Infraest. transp. and other serv. (taxis, buses) ^b^	2.2	4.3	65.1

^a^: Note that it is a weighted sample. ^b^: Note that regarding the theoretical model (questionnaire) in the factor analysis, the dimension “infraest. transp. and other serv.” is divided into two factors (7 and 8).

**Table 9:** Construct validity: association (Pearson correlation) ^a^ between global assessment by dimension, general satisfaction and score of the main questionnaire (*n* = 725).

Variable	Scores by Dimension Calculated with All Items							Total Score
	ACC	CAT	SPA	SPO	LEI	PUB	INF	
Global valuation items ^b^								
ACC	0.84							
CAT		0.91						
SPA			0.81					
SPO				0.85				
LEI					0.9			
PUB						0.76		
INF							0.69	
Satisf. general	0.57	0.62	0.67	0.29	0.4	0.53	0.38	0.7
Satisf. price quality	0.45	0.48	0.62	0.31	0.26	0.35	0.33	0.54

^a^: In the table, Pearson’s linear correlation coefficient (r) is shown. Note that it is a weighted sample and that the cases with NS/NC values of the variables in the first column are excluded (see Table 10 **). In all cases, it was statistically significant (*p* < 0.001). ^b^: ACC: accommodation; CAT: catering; SAP: spa; SPO: sports facilities; LEI: leisure/culture/shopping; PUB: public roads/urban and natural environment; INF: transport infrastructures and other services.

**Table 10:** Construct validity: association (comparison of means) between the variables of future intention and the scores estimated ^a^.

Variable	Scores by Dimension ^b^ Calculated with all Items							Punctuation Total
	ACC	CAT	SPA	SPO	LEI	PUB	INF	
Intent to recommend it								
Yes	5.95 ± 0.86	6.02 ± 1.05	6.01 ± 0.75	3.95 ± 1.71	4.52 ± 1.41	6.00 ± 0.94	4.31 ± 1.22	5.47 ± 0.75
No do not know	5.15 ± 0.99	4.59 ± 1.39	4.86 ± 1.09	2.68 ± 1.25	3.16 ± 1.32	5.00 ± 1.20	3.11 ± 1.29	4.29 ± 0.90
*p*-value ^c^	*p* < 0.001	*p* < 0.001	*p* < 0.001	*p* < 0.001	*p* < 0.001	*p* < 0.001	*p* < 0.001	*p* < 0.001
Intention to return								
Next year/this year	6.03 ± 0.85	6.13 ± 1.03	6.12 ± 0.72	3.93 ± 1.76	4.54 ± 1.45	6.12 ± 0.88	4.32 ± 1.24	5.56 ± 0.73
ever/do not know/never	5.45 ± 0.87	5.25 ± 1.18	5.36 ± 0.92	3.61 ± 1.56	4.05 ± 1.36	5.35 ± 1.09	3.92 ± 1.30	4.85 ± 0.87
*p*-value ^c^	<0.001	<0.001	<0.001	0.019	<0.001	<0.001	<0.001	<0.001

^a^: Based on the 725 users, since they all answered the questions in column 1. The sample sizes are in Table 7 **. The mean ± standard deviation is given in the table. Note that it is a weighted sample. ^b^: ACC: accommodation; CAT: catering; SPA: spa; SPO: sports facilities; LEI: leisure/culture/shopping; PUB: public roads/urban and natural environment; INF: transport infrastructures and other services. ^c^: T-student for independent samples, with weighted sample

## Data Availability

The data presented in this study are available on request from the corresponding author.

## References

[1] De la Hoz-Correa A., Muñoz-Leiva F., Bakucz M. (2018). Past themes and future trends in medical tourism research: A co-word analysis. Tour. Manag..

[2] Anaya-Aguilar R., Anaya-Aguilar C., Bravo-Pérez M. (2017). Balneario: La definición de una tradición milenaria en Andalucía. Rev. Derecho Empres. Soc..

[3] Anaya Aguilar R.M. (2011). Diagnóstico y Tendencias del Turismo de Balnearios en Andalucía.Universidad de Málaga, Servicio de Publicaciones. https://riuma.uma.es/xmlui/handle/10630/5077.

[4] Camisón C., Cruz S., González T. (2006). Gestión de la Calidad: Conceptos, Enfoques, Modelos y Sistemas.

[5] Campón-Cerro A.M., Di-Clemente E., Hernández-Mogollón J.M., Folgado-Fernández J.A. (2020). Healthy Water-Based Tourism Experiences: Their Contribution to Quality of Life, Satisfaction and Loyalty. Int. J. Environ. Res. Public Health.

[6] Consejería de Turismo y Deporte de la Junta de Andalucía (2020). Inicio—Web Oficial de Turismo de Andalucía. https://www.andalucia.org/es/conoce-andalucia/situacion-geografica.

[7] Instituto Estadistica y Cartografia de Andalucia (IECA) (2021). Junta de Andalucía. Padrón Municipal de Habitantes. Cifras Oficiales de Población Municipal | Instituto de Estadística y Cartografía de Andalucía. https://www.juntadeandalucia.es/institutodeestadisticaycartografia/padron/index.htm.

[8] Instituto Estadistica y Cartografia de Andalucia (IECA) (2021). Junta de Andalucia. Encuesta de Coyuntura Turística de Andalucía. Cuarto Trimestre 2019 y año 2020 | Instituto de Estadística y Cartografía de Andalucía. https://www.juntadeandalucia.es/institutodeestadisticaycartografia/turismo/notaprensa.htm.

[9] Consejería de Turismo y Comercio de Andalucía (2015). Turismo de Salud y Bienestar. https://www.juntadeandalucia.es/turismoydeporte/opencms/areas/servicios/centro-documentacion/publicaciones/turismo/Turismo-de-salud-y-bienestar-en-Andalucia-00001/.

[10] Otero Cordones C. (2003). Evaluación de la Calidad de Destinos Turísticos de Soly Playa: Una Aplicación a la Costa del Sol. Universidad de Málaga. http://www.biblioteca.uma.es/bbldoc/tesisuma/16699336.pdf.

[11] Kozak M., Rimmington M. (2000). Tourist Satisfaction with Mallorca, Spain, as an Off-Season Holiday Destination. J. Travel Res..

[12] Cong L.C. (2016). A formative model of the relationship between destination quality, tourist satisfaction and intentional loyalty: An empirical test in Vietnam. J. Hosp. Tour. Manag..

[13] Otero Moreno J., Otero Cordones C. (2004). Evaluación de la calidad de destinos turísticos: El caso de la Costa del Sol. Pap. Econ. Esp..

[14] Cronin J.J., Taylor S.A. (1994). Servperf versus Servqual: Reconciling Performance-Based and Perceptions-Minus-Expectations Measurement of Service Quality. J. Mark..

[15] Maddox R.N. (1985). Measuring Satisfiaction with Tourism. J. Travel Res..

[16] Westbrook R.A. (1980). A Rating Scale for Measuring Product/ Service Satisfaction. J. Mark..

[17] Consejería de Turismo y Deporte de la Junta de Andalucía (2021). www.andalucia.org—Web Oficial de Turismo de Andalucía. https://www.andalucia.org/es/segmentos-turisticos.

[18] Echtner C.M., Ritchie J.R.B. (1993). The Measurement of Destination Image: An Empirical Assessment. J. Travel Res..

[19] Westbrook R.A., Oliver R.L. (1991). The Dimensionality of Consumption Emotion Patterns and Consumer Satisfaction. J. Consum. Res..

[20] Sasser W.E., Olsen R.P., Wyckoff D.D., Harvard University (1978). Graduate School of Business Administration. Management of Service Operations: Text, Cases, and Readings.

[21] Gronroos C., Grönroos C., Gronroos C. (1984). A Service Quality Model and its Marketing Implications. Eur. J. Mark..

[22] Parasuraman A., Zeithaml V.A., Berry L.L. (1985). A Conceptual Model of Service Quality and Its Implications for Future Research. J. Mark..

[23] Parasuraman A., Zeithaml V.A., Berry L.L. (1988). Servqual: A Multiple-Item Scale for Measuring Consumer Perceptions of service quality. J. Retail..

[24] Rust R.T., Zahorik A.J., Keiningham T.L. (1995). Return on Quality (ROQ): Making Service Quality Financially Accountable. J. Mark..

[25] Dabholkar P.A., Thorpe D.I., Rentz J.O. (1996). A measure of service quality for retail stores: Scale development and validation. J. Acad. Mark. Sci..

[26] Brady M.K., Cronin J.J. (2001). Some new thoughts on conceptualizing perceived service quality: A hierarchical approach. J. Mark..

[27] Cronin J.J., Taylor S.A. (1992). Measuring Service Quality: A Reexamination and Extension. J. Mark..

[28] Parasuraman A., Berry L.L., Zeithaml V.A. (1991). Perceived service quality as a customer-based performance measure: An empirical examination of organizational barriers using an extended service quality model. Hum. Resour. Manag..

[29] Alén González M.E. (2006). Comparison of scales for the measurement of perceived quality in thermal spas. Rev. Galega Econ. Publ. Interdiscip. Fac. Cienc. Econ. Empres..

[30] Teas R.K. (1994). Expectations as a Comparison Standard in Measuring Service Quality: An Assessment of a Reassessment. J. Mark..

[31] Quester P.G., Romaniuk S., Wilkinson J.W. (2015). A Test of Four Service Quality Measurement Scales: The Case of the Australian Advertising Industry. Proceedings of the 1995 World Marketing Congress.

[32] Bigné J.E., Andreu L., Gnoth J. (2005). The theme park experience: An analysis of pleasure, arousal and satisfaction. Tour. Manag..

[33] Hu Y., Ritchie J.R.B. (1993). Measuring Destination Attractiveness: A Contextual Approach. J. Travel Res..

[34] Kim S., Yoon Y. (2003). The Hierarchical Effects of Affective and Cognitive Components on Tourism Destination Image. J. Travel Tour. Mark..

[35] Rodríguez-Díaz M., Espino-Rodríguez T.F. (2008). A Model of Strategic Evaluation of a Tourism Destination Based on Internal and Relational Capabilities. J. Travel Res..

[36] Giddens A. (1985). Time, space and regionalisation. Social Relations and Spatial Structures.

[37] Robinson S. (1999). Measuring service quality: Current thinking and future requirements. Mark. Intell. Plan..

[38] Bigné J.E., Sánchez M.I., Sánchez J. (2001). Tourism image, evaluation variables and after purchase behaviour: Inter-relationship. Tour. Manag..

[39] Castro C.B., Armario E.M., Ruiz D.M. (2007). The influence of market heterogeneity on the relationship between a destination’s image and tourist’s future behaviour. Tour. Manag..

[40] Chen C.-F., Tsai D. (2007). How destination image and evaluative factors affect behavioral intentions?. Tour. Manag..

[41] Lee T.-H.H. (2009). A structural model for examining how destination image and interpretation services affect future visitation behavior: A case study of Taiwan’s Taomi eco-village. J. Sustain. Tour..

[42] Streiner D.L., Norman G.R. (2008). Health Measurement Scales: A practical Guide to Their Development and Use.

[43] Landis J.R., Koch G.G. (1977). The Measurement of Observer Agreement for Categorical Data. Biometrics.

[44] Martínez-González M.A., Sánchez-Villegas A., López del Burgo C., Martínez-González M.A., Sánchez-Villegas A., Faulín-Fajardo F.J. (2006). Introducción a los modelos multivariables. Bioestadística Amigable.

[45] Hair J.F., Anderson R.E., Tatham R.L., Black W.C. (1999). Análisis Multivariante.

[46] Norman G.R., Streiner D.L. (1996). Bioestadística. https://scholar.google.com/scholar?hl=es&as_sdt=0%2C5&q=Norman%2C+G.R.%2C+Streiner%2C+DL.%281996%29.+Bioestadística.+Madrid%3A+Mosby%2FDoyma+Libros%2C+S.A.&btnG=.

